# Artificial Intelligence in Aesthetic Dentistry: Is Treatment with Aligners Clinically Realistic?

**DOI:** 10.3390/jcm13206074

**Published:** 2024-10-12

**Authors:** Thomas Mourgues, María José González-Olmo, Luis Huanca Ghislanzoni, Cecilia Peñacoba, Martín Romero-Maroto

**Affiliations:** 1 Department of Orthodontics, Rey Juan Carlos University, 28922 Alcorcón, Madrid, Spain; pix117@hotmail.fr (T.M.); martin.romero@urjc.es (M.R.-M.); 2 Division of Orthodontics, Clinics of Dental Medicine, University of Geneva, 1205 Geneva, Switzerland; luis@3dorthodontist.ch; 3 Department of Psychology, Rey Juan Carlos University, Avda. de Atenas s/n, 28922 Alcorcón, Madrid, Spain; cecilia.penacoba@urjc.es

**Keywords:** SmileView™, artificial intelligence, smile simulation

## Abstract

Smile aesthetics are increasingly prioritized in dental practice, with accurate orthodontic assessment and treatment planning being crucial for optimal outcomes. This study evaluates Invisalign^®^ SmileView™ (SV), an AI-based tool that simulates post-treatment smiles, focusing on its ability to present potential orthodontic outcomes to patients. **Background/Objectives**: This research aims to study whether SV can simulate predictable orthodontic results and if it makes anatomical modifications to the teeth. Additionally, it will evaluate whether SV displays smiles that conform to the orthodontic criteria described in the literature. Finally, the study will analyze whether the software can align the dental with the facial midline. **Methods**: A total of 51 subjects were recruited in Madrid, Spain. The operator took a frontal photograph of the subjects with a social smile (T0), following the application’s instructions. Subsequently, the subjects followed the steps to modify their smile (T1), resulting in a new image of the subject with a different smile. The following variables were collected, analyzed, and compared with the standards defined in the literature: smile width, vertical exposure of the maxillary central incisor, width of the maxillary central and lateral incisors, proportion of the maxillary lateral incisor width to the central incisor, anterior gingival exposure level, position of the upper and lower dental midlines relative to the facial midline. **Results**: 58% of the sample showed dental expansion, with an excessive expansion (>5 mm) observed in 8%. In the maxillary arch, 5.9% of incisors exceeded predictable aligner movement (>1.5 mm), with 3.9% showing excessive extrusion and 2% excessive intrusion. For the lateral incisors, the mesiodistal size was reduced less than 0.5 mm in 31.4% of cases, with excessive interproximal reduction (>0.5 mm) in 5.9%. Additionally, 62.7% of cases would require multidisciplinary treatment due to an increase in size. SV centered the upper midline in 77.9% of these cases. Among the sample, the upper midline was initially centered in 74.5% of subjects, and SV maintained it centered in 84.2% of these subjects. **Conclusions**: SV tends to generate simulations of broader smiles, which are mostly achievable through aligner treatments, from an orthodontic perspective, and showed high predictability regarding the vertical movements of the incisors that can be achieved with aligners. Moreover, it adjusted the mesiodistal size of the upper incisors in its simulations and demonstrated the ability to identify and correct deviations of the dental midlines relative to the facial midline.

## 1. Introduction

Smile aesthetics play a crucial role in contemporary dental practice, as reflected by the increasing demand for more cosmetic and aesthetic procedures from patients [1]. Achieving an optimal aesthetic outcome in oral rehabilitation requires several critical stages, including a preliminary orthodontic assessment, accurate diagnosis, and appropriate treatment planning. These steps are essential in the overall process of dental and facial restoration [2,3]. From an orthodontic perspective, the smile comprises various features, such as the smile line, which is defined as the distance between the upper lip and the maxillary anterior teeth when smiling, and the smile arc, which is the relationship between the curvature of the maxillary anterior teeth and the upper border of the lower lip [4,5]. The number of teeth displayed (the count of visible teeth when smiling), the size of the buccal corridors, the variations between the central and lateral incisal edges of the maxillary incisors, the presence of crowding or diastemas, the overbite, and the relationship between the dental and facial midlines all play a fundamental role in the aesthetics of the smile [3,6,7]. Additionally, aspects such as excessive gingival display when smiling, known as a “gummy smile”, which is not a pathological condition and occurs when more than 3 to 4 mm of gingiva are visible when smiling, must also be considered [8].

There are two main types of smiles: the social smile and the emotional smile. The social smile is deliberately used in social situations to express politeness and kindness, adhering to social conventions. In contrast, the emotional smile is genuine and reflects authentic emotions such as joy and affection. Both types are crucial in our communication and interaction with others [9,10,11].

Invisalign^®^ SmileView™ (SV) is an interactive tool provided by Invisalign^®^ that allows prospective patients to visualize how their smile might improve following treatment with aligners. This technology employs 3D modeling to display the patient’s current smile and simulate the changes that Invisalign aligners can achieve in dental alignment. This way, patients can virtually see potential outcomes before initiating the actual treatment [12]. It is important to note that the smile simulator is based on a vast database of 6 million Invisalign cases, offering a precise simulation of possible dental results. This system uses a machine learning algorithm to achieve this accuracy. The official launch of SV was in 2019. Dr. Rhona Eskander, at the British Dental Conference and Dentistry Show in 2019, mentioned that the primary purpose of SV is to attract patients, thereby increasing treatment acceptance and driving business growth [13].

Regarding the creation of that new smile, there is no information available on the specific orthodontic movements performed by artificial intelligence (AI) to alter the smile, nor is it known if they are biomechanically feasible. To date, no studies have been found that analyze the “aesthetic smile” standards defined by AI or confirm whether it meets the clinical criteria established by the scientific community. Therefore, this research aims to study whether SV can simulate predictable orthodontic results and determine if it makes anatomical modifications to the teeth. Additionally, it will evaluate whether SV displays smiles that conform to the orthodontic criteria described in the literature, including gingival display. Finally, the study will analyze whether the software can align the dental midline with the facial midline.

## 2. Methods

### 2.1. Study Type and Design

A prospective longitudinal cohort study was conducted in two phases: T0 (measurement of smile characteristics in an initial photograph) and T1 (after modifying the smile using the SV application).

### 2.2. Study Population

A power analysis was performed to determine the minimum sample size required for a test with two variables, with a significance level of α ≤ 0.05 and a power of 80%. Based on previous studies, it was established that a 2 mm difference in incisor display would be clinically relevant, and a 3 mm difference in smile width would be statistically significant [14]. The minimum sample size was determined to be 48 subjects.

A total of 51 subjects were recruited in Madrid, Spain, from shopping centers, educational institutions, and offices. Exclusion criteria included visible caries in the smile, missing teeth, periodontal disease, or the presence of any severe facial abnormalities. Additionally, patients whose smiles did not allow clear visualization of the lower incisors, the incisal edges, and the widest (mesiodistal) part of the central and lateral maxillary incisors were also excluded.

### 2.3. Ethical Considerations

The study protocol was reviewed and approved by the Ethics Committee of the Rey Juan Carlos University, Madrid, under internal number (2807202329823). The purpose of the study was explained to the participants, and the confidentiality of the collected information was assured. All patients who agreed to participate in the study signed an informed consent form.

### 2.4. Procedure

**Figure 1:**
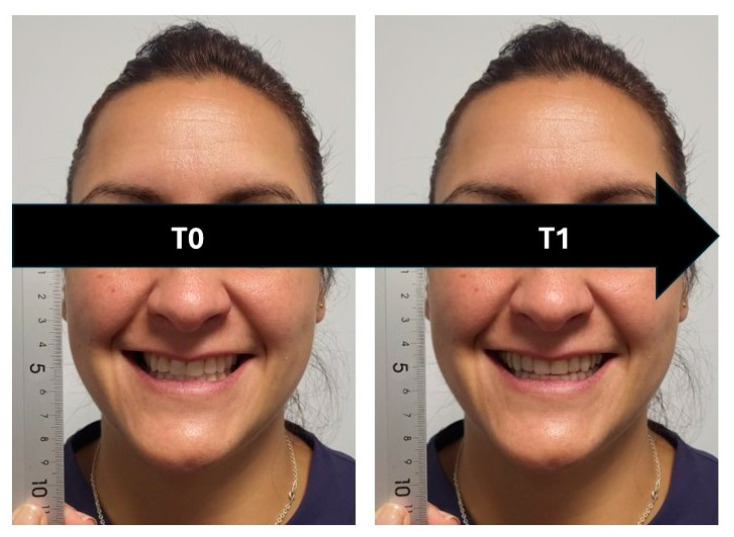
Smile simulation for Subject 1. T0 represents the initial photograph taken by the operator before the simulation. T1 shows the result obtained after processing the photograph through the application.

The operator took a frontal photograph of the subjects with a social smile (T0) following the application’s instructions. Subsequently, the subjects followed the steps to modify their smile (T1) (12), resulting in a new image of the subject with a different smile (Figure 1). In their left hand, the subjects held a millimeter ruler positioned at the height of the smile, allowing for digital calibration of the photographs and enabling precise measurement of the smile structures.

### 2.5. Instruments and Measurements

To ensure reliability and reproducibility, the same operator measured the distances twice using the same photograph, with the second measurement taken several days later to minimize bias. NemoCeph software (NemoStudio20) was used to measure the following variables in millimeters during the T0 phase (initial social smile) and the T1 phase (social smile modified by the SV platform 2024). A millimeter ruler with a precision of 0.01 mm was used within the NemoCeph software (NemoStudio20) for the accurate calibration and measurement of smile characteristics.

Smile width (in millimeters): The width was calculated based on the most lateral visible teeth in the smile [3]. This measurement allowed for the evaluation of the predictability of orthodontic movements involving dentoalveolar expansion or compression. Various studies evaluating dental arch expansion suggest that, to minimize the risk of gingival recession and ensure a high planned movement predictability in the initial setup, applying appropriate biomechanics, the arch width expansion should be limited to a maximum of 2 to 3 mm per quadrant [15,16,17,18]. Therefore, a total expansion of 5 mm was considered to be realistic and predictable movement with aligners.

Vertical exposure of the maxillary central incisor (in millimeters): A randomly selected maxillary central incisor was used to measure the distance from the incisal edge of the tooth to the marginal gingiva or the lowest part of the lip (if gingiva was not visible). The vertical movement of incisors is the most challenging aspect to manage with aligners. In a study addressing open bite cases, an average extrusion of the maxillary incisor of 1.45 ± 0.89 mm was achieved [18]. Other studies have demonstrated that the average intrusion of incisors in deep bite cases ranges from 0.75 to 1.50 mm [19,20]. Therefore, realistic movement in this study was considered to be up to 1.5 mm of intrusion and 1.5 mm of extrusion.

Width of the maxillary central and lateral incisors (in millimeters): This measurement was taken from the most aligned side. In cases of crowding, the mesiodistal width of a tooth can appear artificially reduced in a 2D image, such as a photograph. To avoid this distortion and ensure more accurate measurements, we selected the tooth on the side where the alignment was closest to ideal. In orthodontics, modifications to the mesiodistal size of a tooth involve a reconstruction or reduction in enamel (interproximal reduction, IPR). The acceptable limit for IPR is determined to be 0.25 mm per side [21]. Enamel reduction exceeding 0.25 mm per side is considered “inappropriate” due to the thickness of the enamel layer and the need to preserve the tooth’s structural integrity. The sample was divided into three groups based on changes in incisor size: cases requiring multidisciplinary treatment (where SV increased tooth width), cases with IPR > 0.25 mm per side, and cases that could be treated solely with orthodontics and aligners (IPR between 0 and 0.25 mm per side).

Proportion of the maxillary lateral incisor width to the central incisor (in percentage): The proportion between the width of the central and lateral incisors was calculated using the previously collected mesiodistal measurements, and the sample was divided into two groups (greater than or less than 0.62) according to the “golden proportion” proposed by Lombardi in 1973 and later developed by Levin [22].

Anterior gingival exposure level (in millimeters): Measured apically to the crown of the maxillary central incisor; the side with the most exposure was selected. According to the literature, aesthetic gingival exposure is considered to be between 1 and 3 mm (24). The sample was divided into two groups: subjects with more than 2 mm of gingival exposure (tending towards a gummy smile) and subjects with less than 2 mm, as described in the study by Rizzi et al. [23].

Position of the upper and lower dental midlines relative to the facial midline (centered/not centered): For diagnosing dental midlines, the facial midline reference point was a straight line passing through the glabella and subnasal point [24]. The distance from the upper and lower dental midlines was calculated.

### 2.6. Statistical Analysis

The data collected were analyzed using SPSS version 28.0. Measurement reproducibility was evaluated through Pearson’s correlation coefficient (r) and the intraclass correlation coefficient (ICC). Descriptive statistics were used to assess the frequency of the following variables: smile width, vertical exposure of the labial surface of the maxillary central incisor, and the mesiodistal width of the incisors, as well as the dental midlines. To assess changes in the ratio of the maxillary lateral incisor width to the central incisor and anterior gingival exposure from T0 to T1, a paired *t*-test was conducted. Significance levels were established at 0.05.

## 3. Results

### 3.1. Method Error

The reproducibility of the measurements was evaluated using Pearson’s correlation coefficient (r) and the intraclass correlation coefficient (ICC). A Pearson coefficient greater than 0.9 was obtained for variables such as smile width, vertical exposure of the maxillary central incisor, the proportion between the maxillary lateral incisor width to the central incisor, and the distance between incisal edges. The other variables showed a Pearson coefficient greater than 0.8. The ICC was above 0.8 for all evaluations, indicating high reproducibility.

### 3.2. Smile Width

**Figure 2:**
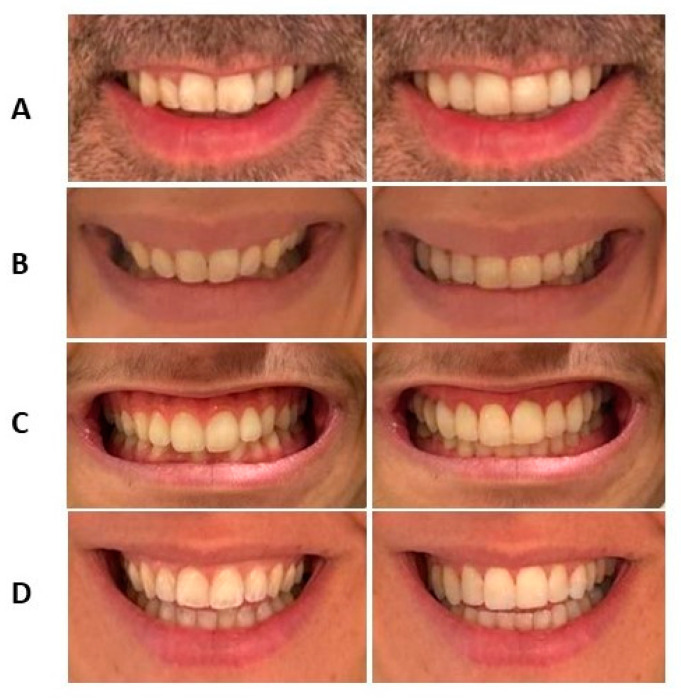
Smile simulation using the SV software. The images taken at T0 are shown on the left, and those taken at T1 are on the right. (**A**). Case of mesiodistal changes in the maxillary incisors. (**B**). Increase in smile width. (**C**). Reduction in anterior gingival exposure. (**D**). Correction of a deviation in the lower dental midline.

Regarding smile width, 58% of the sample showed dental expansion (CI: 58.0 ± 13.77%). Excessive expansion (>5 mm) was observed in 8% of cases (CI: 8.0 ± 7.84%). See Figure 2.

### 3.3. Vertical Exposure of the Maxillary Central Incisor

In the maxillary arch, 5.9% (*n* = 3) of the incisors exceeded the range of predictable movement with aligners (>1.5 mm). Excessive extrusion occurred in 3.9% (*n* = 2) of cases and excessive intrusion occurred in 2% (*n* = 1) of cases.

### 3.4. Mesiodistal Width of the Maxillary Central Incisors

For the central incisors, the mesiodistal size was reasonably reduced in 51% (*n* = 26) of cases, with excessive interproximal reduction in 27.5% (*n* = 14). Additionally, 21.6% (*n* = 11) of cases would require multidisciplinary treatment due to an increase in size (Table 1). See Figure 2.

### 3.5. Mesiodistal Width of the Maxillary Lateral Incisor

For the lateral incisors, the mesiodistal size was reasonably reduced in 31.4% (*n* = 16) of cases, with excessive interproximal reduction in 5.9% (*n* = 3). Additionally, 62.7% (*n* = 32) of cases would require multidisciplinary treatment due to an increase in mesiodistal size (Table 2). See Figure 2.

### 3.6. Mesiodistal Proportion of Maxillary Central Incisor to Maxillary Lateral Incisor

Initially, 29.4% of the sample had a discrepancy due to either an oversized central incisor or an undersized lateral incisor (<0.62). SV adjusted the proportion to 0.71 ± 0.07, even within the group with a proportion >0.62, with no significant differences (t = 0.044, *p* = 0.483).

### 3.7. Anterior Gingival Exposure

Initially, 11.8% of the sample presented a gummy smile. SV tended to reduce gingival exposure in both groups, with a greater reduction observed in the gummy smile group (0.36 ± 0.42 mm vs. 0.09 ± 0.64 mm), although the differences were not statistically significant (t = 1.397, *p* = 0.09). See Figure 2.

### 3.8. Upper and Lower Dental Midlines

The upper dental midline was deviated from the facial midline in 25.5% of subjects (*n* = 13). SV centered the upper midline in 77.9% of these cases. Among the sample, the upper midline was initially centered in 74.5% (*n* = 38) of subjects, and SV maintained it centered in 84.2% of these subjects.

The lower dental midline was deviated from the facial midline in 39.2% of subjects (*n* = 20). SV centered the lower midline in 65% of these cases. The lower midline was initially centered in 60.8% (*n* = 31) of subjects, and SV maintained it centered in 67% of these subjects. See Figure 2.

## 4. Discussion

This research aimed to analyze the outcome of the SmileView™ (SV) simulation in relation to established orthodontic standards. Regarding the transverse dimension, it is well-known that buccal corridors negatively impact smile aesthetics [25]. SV, following the Damon system philosophy of broad smiles, tends to propose expansions in 58.8% of the cases studied. Invisalign^®^, through SV, seeks to offer an aesthetically pleasing smile, proposing realistic expansions (<5 mm) in 87.5% of cases. However, aligner treatment has limitations to achieving transverse expansion. Studies indicate that only 61% to 70.88% of the planned expansion is achieved, depending on whether the dentition is mixed or permanent [26,27]. Factors such as the patient’s age and the initial torque of the posterior teeth influence the success of expansion with aligners. In some cases, the use of expanders or palatal disjunctors may be necessary before treatment. Indeed, many studies describe only coronal buccal movement in cases of expansion [17].

Regarding the exposure of the maxillary incisors, SV is conservative, significantly modifying (>1.5 mm) the vertical position of the central incisor in only 5.9% of cases. This reflects the limitations of aligners in performing intrusion/extrusion movements, which have low predictability (29.6% for anterior extrusion and 35% for anterior intrusion) [28]. The effectiveness of orthodontic treatment is influenced by various individual factors that must be carefully assessed before starting the process. It is crucial to measure the initial inclination of the incisor in relation to the bone to avoid having the root move into the cortical bone, which is denser and complicates dental movement during orthodontic treatment. Additionally, other anatomical limitations must be considered, such as the proximity between the tooth root and the maxillary sinus. This proximity can lead to bone remodeling, potentially compromising both tooth stability and periodontal support [29]. It has been observed that proper “staging” during the initial setup can improve predictability [30]. On the other hand, there are alternative methods to influence incisal exposure without moving the anterior teeth, such as changes in molar positioning that affect mandibular rotation. Moreover, relative vertical movements, which are expressions of dental torque, can be combined with other techniques.

When analyzing modifications in the mesiodistal width of the upper incisors, a significant difference was observed in the outcomes of multidisciplinary cases between the central and lateral incisors (21.6% and 62.7%, respectively). This difference could be attributed to the high incidence of microdontia in the lateral incisors. In a study conducted in 2022, it was found that 38.8% of subjects presented with microdontia in at least one of the two maxillary lateral incisors [31]. Traditionally, orthodontists decide whether to maintain or close the diastemas caused by microdontia based on malocclusion criteria such as overjet and Angle’s classification. However, artificial intelligence (AI) appears to favor the reconstruction of the incisor to maintain the ideal dental proportion. This decision would require the intervention of a dental aesthetics specialist and involve additional costs, potentially disregarding the orthodontic criteria mentioned earlier.

In orthodontic practice, significant enamel reduction (greater than 0.5 mm) is employed as a therapeutic technique in cases of macrodontia, where the teeth are excessively large. However, macrodontia is relatively rare in the general population (0.03–1.9%), which does not justify the high percentage observed in the studied sample (27.5% for the central incisor). It is hypothesized that SV may be compensating for the presence of microdontic lateral incisors by adjusting the mesiodistal size of the central incisor to maintain an appropriate dental proportion. This hypothesis highlights the limitations of AI-based diagnostics, which may overlook important epidemiological factors. Another plausible explanation could be the presence of triangular-shaped crowns, observed in 8% of Caucasian subjects [32]. This morphology shifts the contact point towards the incisal edge, creating “black triangles”, a feature associated with dental aging. However, this characteristic is more commonly found in the lower arch. In such cases, the typical orthodontic treatment involves significant reshaping of the crown at the incisal level, followed by the closure of the resulting diastema.

The analysis of the proportion between the central and lateral incisors revealed that SmileView™ (SV) tends to adjust this ratio to 0.72. This result aligns with a study by Kantrong et al., who found a mean ratio of 0.72 in their sample, with an aesthetic preference for a proportion of 0.70 [33]. However, there is no universal consensus, as other studies suggest that the “golden ratio” should be 0.66 [34]. The discrepancies between the results of different studies could be attributed to the variety of research methodologies employed. Factors such as different measurement techniques for data acquisition and analysis, sample size, ethnic groups studied, and the inclusion of individuals with previous orthodontic treatment may significantly contribute to these differences. Artificial intelligence seems programmed to adjust incisor proportions, but it may not fully account for actual proportions due to inherent biases. For example, in cases of malocclusion, such as incisor rotation, anterior crossbite, or open bite, the measurements would be affected. As a result, the program cannot adapt the final (post-treatment) smile based on the initial situation, but rather tends to adjust all smiles to a predetermined “golden ratio”. This process has limitations and may create unrealistic expectations in patients regarding the cost of treatment. Achieving the simulation’s result often requires the involvement of other dental specialties, which can significantly increase the final treatment’s price.

SV tends to reduce anterior gingival exposure across all analyzed groups, although the difference is minimal and not statistically significant. The reduction is more noticeable in the gummy smile group (0.36 ± 0.42), while in the group without excessive exposure, the reduction is smaller (0.09 ± 0.64). However, this tendency of AI to reduce gingival exposure could have negative effects on patients without gummy smile issues, potentially worsening the smile’s aesthetics, as a slight gingival display is often associated with a more youthful and healthy appearance. Furthermore, the absence of gingival display can result in a flat or straight smile arc, which may be perceived as less dynamic and attractive compared to smiles that show a slight curve or some gingival tissue [35]. The causes of a gummy smile require an orthodontic and/or multidisciplinary diagnosis to determine the appropriate therapeutic approach. Factors such as a short lip, hypermobile lip activity, altered passive eruption, vertical maxillary excess, and gingival hyperplasia are not corrected with conventional orthodontic treatments. These aspects must be considered by the orthodontist before initiating treatment to achieve optimal results [8]. For these reasons, AI does not significantly modify anterior gingival exposure, which is realistic in most cases when using aligners alone.

In the analysis of dental midlines, SV demonstrates greater accuracy in the upper incisors. This is primarily because, during a smile, the upper incisors are more visible than the lower ones, making it easier for the artificial intelligence to locate them in relation to the face. On the other hand, SV’s effectiveness in correcting the lower midline is less reliable. This could be attributed to two main factors: overbite and a significant tendency for lower crowding. Lower crowding, in particular, can make it difficult for the AI system to accurately identify the dental midline, resulting in lower precision in its proposed corrections to the lower arch.

Among the practical implications of this research, it can be stated that SV generates smiles that align with the aesthetic standards established in the literature, making it a valuable tool for orthodontists to promote their treatments. However, it may create confusion among patients by modifying dental proportions without considering the initial anatomical variations. It is crucial to clarify to patients that this is a simulation and not a definitive treatment plan.

With the integration of artificial intelligence in medicine, questions arise about its limitations. In the context of smile simulation in orthodontics, SV might create unrealistic expectations, as orthodontic practice has its constraints. Some dental clinics already include disclaimers on their websites to prevent possible misunderstandings, such as, “It’s important to note that this is purely a simulation, albeit a lifelike one, and the final treatment outcome may differ”.

For orthodontists, SV can be useful in planning dental arch expansions and making decisions to manage cases of microdontia. It would be interesting to study potential patients’ reactions to these simulations to assess their effectiveness as a persuasive tool.

In the future, SV could incorporate 3D imaging, such as cone beam computed tomography (CBCT), soft tissue imaging, and stereolithographic (STL) models of the patient’s mouth, to enhance the accuracy of simulations. It would also be valuable for future versions of the Invisalign SmileView™ platform to incorporate the possibility of human intervention after generating the AI simulation. Allowing the operator to make manual adjustments could better tailor the simulated smile to the patient’s unique dental and facial characteristics. This flexible feature could significantly improve patient satisfaction and treatment predictability.

Given that the use of two-dimensional images in a study analyzing orthodontic factors limits the precision of the results, this research has certain limitations. Dental proportions may be inaccurate if the teeth are not properly aligned, and the U-shaped form of the dental arch implies a depth of field that is not captured in 2D. Although the study’s photographs were taken under optimal conditions, SV is designed to be used with the front camera of a mobile phone, which could result in varying angles and, consequently, different outcomes than those obtained in the study.

## 5. Conclusions

1. SV tends to generate simulations of broader smiles that, from an orthodontic perspective, are mostly achievable through aligner treatments.
2. SV simulations show high predictability regarding the vertical movement of incisors that can be achieved with aligners.
3. SV adjusts the mesiodistal size of the upper incisors in its simulations.
4. The software modifies the mesiodistal proportion of the upper incisors, aiming for a “golden ratio” of 0.72, which implies alterations to dental dimensions.
5. SV’s artificial intelligence does not make significant changes to gingival exposure, although a slight improvement in this aspect is observed.
6. SV demonstrates the ability to identify and correct deviations in the dental midlines relative to the facial midline. However, there is a greater margin of error in the proposed corrections for the lower arch.

## Figures and Tables

**Table 1:** Change in Mesiodistal Size of the Central Incisor at T1.

	Frequency	Percentage (%)	Cumulative Percentage (%)
Enamel Reduction (Orthodontic)	26	51.0	51.0
Multidisciplinary Case	11	21.6	72.5
Excessive Enamel Reduction	14	27.5	100.0
Total	51	100.0	

**Note:** Multidisciplinary case (>0 mm), excessive enamel reduction (<−0.5 mm), enamel reduction (orthodontic) [−0.5 to 0 mm].

**Table 2:** Change in Mesiodistal Size of the Lateral Incisor at T1.

	Frequency	Percentage (%)	Cumulative Percentage (%)
Enamel Reduction (Orthodontic)	16	31.4	31.4
Multidisciplinary Case	32	62.7	94.1
Excessive Enamel Reduction	3	5.9	100.0
Total	51	100.0	

**Note:** Multidisciplinary case (>0 mm), excessive enamel reduction (<−0.5 mm), enamel reduction (orthodontic) [−0.5 to 0 mm].

## Data Availability

The data that support the findings of this study are available on request from the corresponding author.
